# Tim-4 functions as a scavenger receptor for phagocytosis of exogenous particles

**DOI:** 10.1038/s41419-020-02773-7

**Published:** 2020-07-23

**Authors:** Chanhyuk Min, Jeongjun Park, Gayoung Kim, Hyunji Moon, Sang-Ah Lee, Deokhwan Kim, Byeongjin Moon, Susumin Yang, Juyeon Lee, Kwanhyeong Kim, Hyeokjin Cho, Jihwan Park, Dae-Hee Lee, Gwangrog Lee, Daeho Park

**Affiliations:** 1 School of Life Sciences, Gwangju Institute of Science and Technology, Gwangju, 61005 Republic of Korea; 2 Laboratory for cell mechanobiology, Gwangju Institute of Science and Technology, Gwangju, 61005 Republic of Korea; 3 Department of Marine Food Science and Technology, Gangneung-Wonju National University, Gangneung, 25456 Republic of Korea

**Keywords:** Apoptosis, Extracellular signalling molecules

## Abstract

The phosphatidylserine (PS) receptor Tim-4 mediates phagocytosis of apoptotic cells by binding to PS exposed on the surface of these cells, and thus functions as a PS receptor for apoptotic cells. Some of PS receptors are capable of recognizing other molecules, such as LPS on bacteria, besides PS on apoptotic cells. However, it is unclear whether Tim-4 perceives other molecules like the PS receptors. Here, we report that Tim-4 facilitates the phagocytosis of exogenous particles as well as apoptotic cells. Similar to the process that occurs during Tim-4-mediated efferocytosis, the uptake of exogenous *E. coli* and *S. aureus* bioparticles was promoted by overexpression of Tim-4 on phagocytes, whereas phagocytosis of the bioparticles was reduced in Tim-4-deficient cells. A truncation mutant of Tim-4 lacking the cytoplasmic tail promoted phagocytosis of the particles, but a mutant lacking the IgV or the mucin domain failed to enhance phagocytosis. However, expression of Tim-4^AAA^ (a mutant form of Tim-4 that does not bind phosphatidylserine and does not promote efferocytosis) still promoted phagocytosis. Tim-4-mediated phagocytosis was not blocked by expression of the phosphatidylserine-binding protein Anxa5. Furthermore, binding of lipopolysaccharide (LPS), which is found in the outer membrane of Gram-negative bacteria, was higher in Tim-4-overexpressing cells than in Tim-4-deficient cells. In summary, our study suggests that Tim-4 acts as a scavenger receptor and mediates phagocytosis of exogenous particles in a phosphatidylserine-independent manner.

## Introduction

Scavenger receptors are a large family of cell-surface receptors that participate in a broad range of biological functions. Originally, they were categorized on the basis of their ability to bind to modified low-density lipoproteins (LDL). They recognize and remove a diverse variety of ligands, including endogenous and modified host-derived molecules (DAMPs, damage-associated molecular patterns) and microbial pathogens (PAMPs, pathogen-associated molecular patterns)^1,2^. Phosphatidylserine (PS) exposed on the cell surface could function as an endogenous DAMP^3^. Lipids across the plasma membrane are asymmetrically distributed. PS is exclusively located on the inner leaflet of the plasma membrane, and this asymmetrical distribution of PS across the membrane is maintained by the action of scramblases and flippases^4,5^. During apoptosis, the distribution of PS is disrupted, and it is exposed on the outer leaflet of the membrane and oxidized^6,7^. In this way, PS exposed on the cell surface functions as an eat-me signal, and is indispensable for phagocytosis of apoptotic cells, called efferocytosis^8^.

Exposure of PS on the cell surface is used for the specific recognition of apoptotic cells by phagocytes. In efferocytosis, the PS on the cell surface is recognized by various receptors known as engulfment receptors^9^. A subset of these receptors, called PS receptors, directly binds to PS^10–13^, while the others indirectly recognize it through bridging molecules such as Gas6 and Mfge8^14–17^. Many PS receptors have been categorized as, or suggested to be, members of the scavenger receptor family because they recognize both PS and PAMPs^18^. For example, BAI1 recognizes PS on apoptotic cells as well as LPS on Gram-negative bacteria through its TSR domain, and thus enhances clearance of apoptotic cells and Gram-negative bacteria^11,19^. In addition, Tim-1 (also called KIM-1, kidney injury molecule-1, or HAVCR1, hepatitis A virus cellular receptor 1) recognizes both PS on apoptotic cells and oxidized LDL^20–22^, and Stablilin2 also functions as a receptor for apoptotic cells and bacteria^12,23,24^.

Tim-4 (T-cell immunoglobulin and mucin domain-containing 4) is one of the best-characterized PS receptors. Tim-4 was initially identified as a protein that interacted with Tim-1 to regulate T-cell proliferation^25^. Tim-4 consists of a relatively short cytoplasmic tail and an extracellular region containing an IgV and a mucin domain^26^. The cytoplasmic tail of Tim-4 is dispensable for signal transduction in efferocytosis, since a truncation mutant of Tim-4 lacking the cytoplasmic tail and the transmembrane domain still promotes efferocytosis^27^. In other words, Tim-4 tethers apoptotic cells to phagocytes during efferocytosis, but does not transduce signals itself. It is suggested that Tim-4 forms a trimeric complex with fibronectin 1 (Fn1) and integrins, which enables efficient recognition of apoptotic cells and mediates their ingestion through the action of integrins^28^. In contrast to the cytoplasmic tail, the IgV domain of Tim-4, which binds to PS on apoptotic cells, is essential for Tim-4 to function as a PS receptor. Mutation of key residues in Tim-4 necessary for PS recognition completely nullifies Tim-4-mediated efferocytosis^29^. Besides the role of Tim-4 in efferocytosis, Tim-4 is involved in mediating the infection of pathogens, such as some enveloped viruses and *Listeria monocytogenes*. The pathogens mimic apoptotic cells by exposing PS on viral envelopes^30,31^ or on membrane-derived vesicles containing the bacteria^32^, and thus could exploit Tim-4 in order to be internalized into host cells or to spread to other cells. Thus, Tim-4 actively mediates phagocytosis of host-derived apoptotic cells, as well as the passive infection of some pathogens into cells in a PS-dependent manner. Nevertheless, it is uncertain if and how Tim-4 mediates phagocytosis of other exogenous particles such as bacteria other than apoptotic cells.

In this study, we evaluated whether Tim-4 mediates phagocytosis of *E. coli* and *S. aureus* bioparticles, and if Tim-4-mediated phagocytosis is dependent upon PS. We found that the level of phagocytosis was dependent upon the expression level of Tim-4 and the number of bioparticles able to bind to Tim-4. Phagocytosis mediated by Tim-4^AAA^, a mutant of Tim-4 that does not bind to PS, was commensurate with that mediated by wild-type Tim-4, and Tim-4-mediated phagocytosis of the particles was not blocked by expression of Anxa5, a PS-binding protein. In addition, phagocytosis mediated by a Tim-4 mutant without the cytoplasmic tail and the transmembrane domain was comparable to phagocytosis mediated by wild-type Tim-4, whereas a Tim-4 truncation mutant without the IgV or the mucin domain did not promote phagocytosis of the bioparticles. Collectively, our observations suggest that Tim-4 acts as a scavenger receptor for exogenous bioparticles independently of PS to facilitate their phagocytosis.

## Results

### Tim-4 enhances phagocytosis of exogenous particles as well as apoptotic cells

**Fig. 1:**
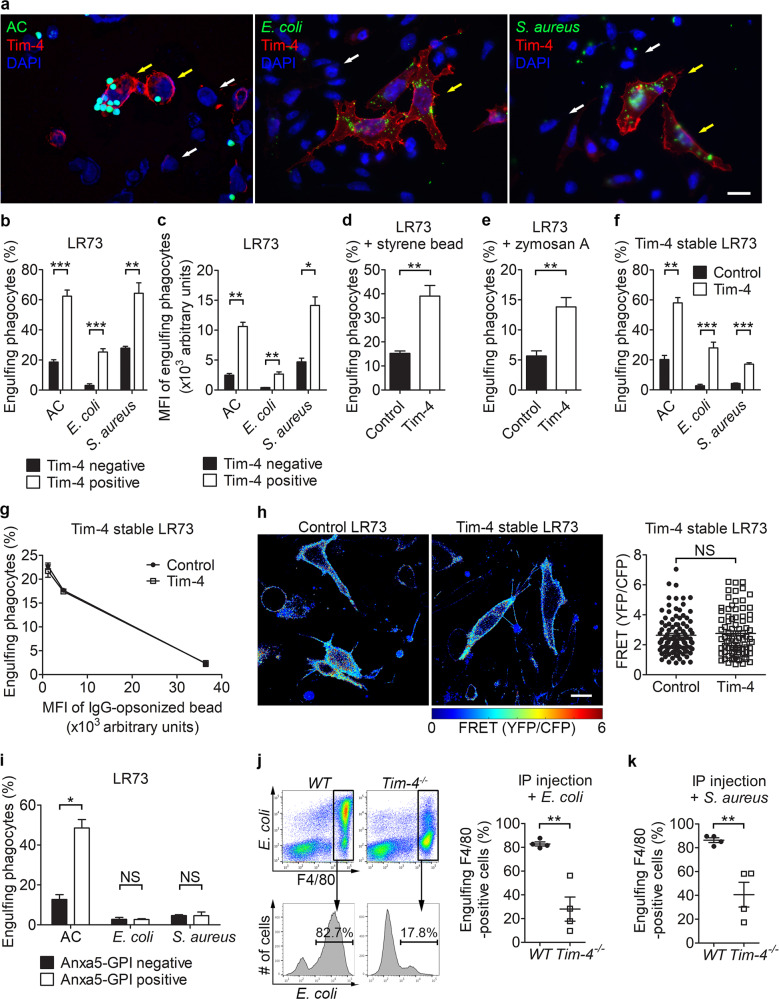
Tim-4 promotes phagocytosis of exogenous particles. **a**–**c** LR73 cells transfected with HA-Tim-4 were incubated with TAMRA-labeled apoptotic thymocytes, FITC-labeled *E. coli*, or *S. aureus* bioparticles for 2 h, extensively washed with ice-cold PBS, stained with anti-HA antibody, and observed using confocal microscopy (**a**) or flow cytometry (**b**, **c** *n* = 3). TAMRA- or FITC-positive cells were considered to be phagocytes engulfing apoptotic thymocytes, or *E. coli* or *S. aureus* bioparticles, respectively. Yellow arrows indicate Tim-4-positive cells, and white arrows indicate Tim-4-negative cells. Scale bar, 10 µm. **d**, **e** Phagocytosis of red fluorescence-labeled polystyrene beads (carboxylate-modified beads) (**d** *n* = 3) or zymosan A (**e** *n* = 3) by LR73 cells transfected with HA-Tim-4 was analyzed as in (**b**). **f** LR73 cells stably expressing Tim-4 were incubated with the *E. coli* or *S. aureus* bioparticles for 2 h, and engulfing phagocytes were analyzed using flow cytometry (*n* = 4). **g** The indicated cells were incubated with FITC-labeled IgG-coated streptavidin beads for 2 h and analyzed by flow cytometry. **h** LR73 cells stably expressing Tim-4 or control cells were transfected with Raichu-Rac1, and FRET was measured (99 cells for control-stable cells, 84 cells for Tim-4-stable cells). **i** Phagocytosis of the indicated targets by LR73 cells transfected with HA-Anxa5-GPI was evaluated as in (**b**) (*n* = 3). Scale bar, 10 μm. **j**, **k** FITC-labeled *E. coli* (**j**) or *S. aureus* (**k**) bioparticles were intraperitoneally injected into *WT* or *Tim-4*^*−/−*^ mice, and then, 20 min after injection, the mice were sacrificed, and peritoneal exudates were stained with anti-F4/80 antibody. F4/80- and FITC-positive cells were considered to be phagocytes engulfing *E. coli* (**j** four mice per each sample) or *S. aureus* (**k** four mice per each sample) particles. All data are shown as the mean ± standard error of mean. Images are representative of three independent experiments. *P < 0.05, **P < 0.01, ***P < 0.001. NS not significant, AC apoptotic cells.

A number of PS receptors perceive not only PS on apoptotic cells but also other molecules on foreign substances to phagocytose them^18^. However, it is not known whether Tim-4 can recognize bioparticles other than apoptotic cells, or whether phagocytosis of other recognized particles is dependent upon PS on the surface of these molecules. To test this, LR73 cells transiently overexpressing Tim-4 were incubated with fluorescently labeled *E. coli* and *S. aureus* bioparticles or apoptotic cells, and then phagocytosis of the bioparticles or apoptotic cells by LR73 cells was evaluated by confocal microscopy. As expected, Tim-4-positive cells contained more apoptotic cells than Tim-4-negative cells. Interestingly, Tim-4-positive cells also possessed more *E. coli* or *S. aureus* particles than Tim-4-negative cells (Fig. 1a). We also analyzed phagocytosis of the bioparticles using flow cytometry. Similarly, phagocytosis of the bioparticles by LR73 cells overexpressing Tim-4 was superior to that by control cells, as measured by the percentage and the MFI (mean fluorescence intensity, an indicator of the relative number of bioparticles per cell) of LR73 cells that engulfed the bioparticles (Fig. 1b, c). In addition, we tested whether Tim-4 could promote the phagocytosis of zymosan A, a glucan found on the surface of yeast, or nonbioparticles such as carboxylate-modified polystyrene beads. Tim-4-overexpressing cells robustly promoted the phagocytosis of carboxylate-modified polystyrene beads or zymosan A (Fig. 1d, e). The effects of Tim-4 overexpression on the phagocytosis of *E. coli* or *S. aureus* particles were confirmed in LR73 cells stably expressing Tim-4 (Fig. 1f). Phagocytosis of *E. coli* or *S. aureus* bioparticles was not due to an artifact of Tim-4 overexpression on the cell surface because overexpression of Tim-4 neither promoted phagocytosis of IgG-opsonized beads nor altered the basal level of Rac1 activation, which was confirmed by fluorescence resonance energy transfer (FRET) using Raichu-Rac1, a Rac1 biosensor (Fig. 1g, h). In addition, overexpression of Anxa5-GPI, an artificial tethering receptor that binds to PS on apoptotic cells^33^, promoted phagocytosis of apoptotic cells, but did not enhance phagocytosis of *E. coli* or *S. aureus* bioparticles (Fig. 1i).

Next, we evaluated the effects of Tim-4 on phagocytosis of *E. coli* or *S. aureus* particles in vivo. Due to the predominant expression of Tim-4 on peritoneal macrophages and its role in mediating PS-dependent efferocytosis in these cells, phagocytosis of the bioparticles by peritoneal macrophages derived from wild-type (*WT*) or *Tim-4*^*−/−*^ mice was evaluated. We injected *E. coli* or *S. aureus* particles into peritonea of *WT* and *Tim-4*^*−/−*^ mice, and measured phagocytosis of *E. coli* or *S. aureus* particles by peritoneal macrophages. Peritoneal macrophages derived from *Tim-4*^*−/−*^ were less efficient at phagocytosing *E. coli* or *S. aureus* bioparticles than those from *WT* mice, as measured by the percentage of cells engulfing the bioparticles (Fig. 1j, k). These data imply that Tim-4 functions as a receptor for the phagocytosis of the bioparticles.

### Tim-4 mediates binding of the bioparticles to phagocytes

**Fig. 2:**
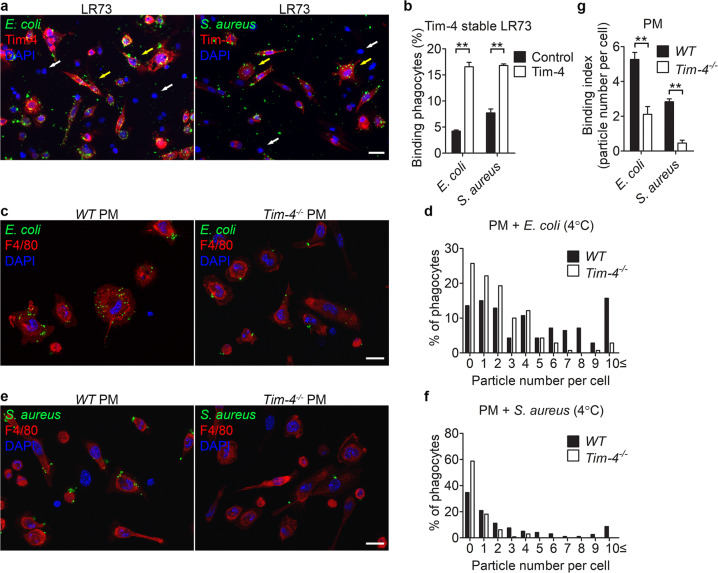
Phagocytes bind to the bioparticles in a Tim-4-dependent manner. **a** LR73 cells transfected with HA-Tim-4 were incubated with *E. coli* or *S. aureus* bioparticles at 4 °C for 2 h, stained with anti-HA antibody, and observed using confocal microscopy. Yellow arrows indicate Tim-4-positive cells, and white arrows indicate Tim-4-negative cells. Scale bar, 20 µm. **b** LR73 cells stably expressing Tim-4 were incubated with *E. coli* or *S. aureus* bioparticles at 4 °C for 2 h, and then binding of these particles to phagocytes was analyzed using flow cytometry (*n* = 3). **c**–**f** Peritoneal macrophages derived from *WT* or *Tim-4*^*−/−*^ mice were incubated with FITC-labeled *E. coli* (**c**, **d** 140 cells for *WT*, 141 cells for *Tim-4*^*−/−*^) or *S. aureus* (**e**, **f** 196 cells for *WT*, 146 cells for *Tim-4*^*−/−*^) bioparticles at 4 °C for 20 min, stained with anti-F4/80 antibody, and observed using fluorescent microscopy. The number of bound targets on phagocytes was quantified (**d**, **f**). Scale bar, 10 µm. **g** The average number of bound targets per phagocyte was calculated from (**c**, **e**). All data are shown as the mean ± standard error of mean. Images are representative of at least three independent experiments. **P < 0.01. NS not significant, PM peritoneal macrophages.

Tim-4 augments binding of apoptotic cells to phagocytes, resulting in enhanced phagocytosis of apoptotic cells. We hypothesized that Tim-4 overexpression or depletion alters the binding of *E. coli* or *S. aureus* particles to phagocytes. To validate this, LR73 cells transiently overexpressing Tim-4 were incubated with *E. coli* or *S. aureus* particles at 4 °C, which allows binding but prevents internalization of the particles. Confocal microscopy results suggested that Tim-4-positive cells bound to more *E. coli* or *S. aureus* particles than Tim-4-negative cells (Fig. 2a). Flow cytometry results from LR73 cells stably expressing Tim-4 confirmed that a higher percentage of LR73 cells stably expressing Tim-4 bound to *E. coli* or *S. aureus* particles than control cells (Fig. 2b). Next, peritoneal macrophages derived from *Tim-4*^*−/−*^ mice were incubated with *E. coli* or *S. aureus* particles at 4 °C. Peritoneal macrophages derived from *Tim-4*^*−/−*^ mice bound to the bioparticles less efficiently than those derived from *WT* mice: a higher percentage of peritoneal macrophages derived from *WT* mice had a high number of bound *E. coli* or *S. aureus* particles than those derived from *Tim-4*^*−/−*^ mice (Fig. 2c–f) and binding index, showing that the number of bound *E. coli* or *S. aureus* particles per phagocyte was about 2.5- or 4-fold higher, respectively, in peritoneal macrophages derived from *WT* mice than in those derived from *Tim-4*^*−/−*^ mice (Fig. 2g). These results suggest that Tim-4 functions as a receptor for *E. coli* or *S. aureus* particles, and that Tim-4 mediates binding of the bioparticles to phagocytes, which enhances phagocytosis of the bioparticles.

### Tim-4 recognizes the bioparticles in a PS-independent manner

**Fig. 3:**
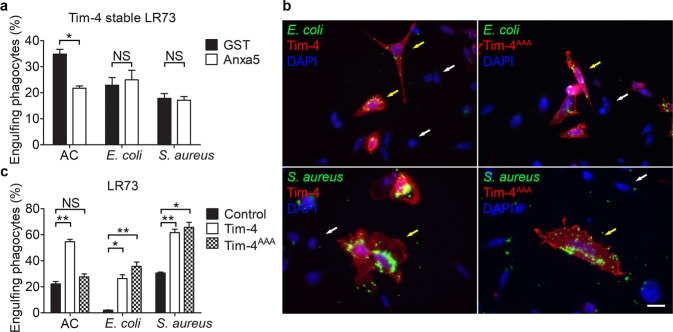
The recognition of PS by Tim-4 is dispensable for Tim-4-mediated phagocytosis of the bioparticles. **a** LR73 cells stably expressing Tim-4 were incubated with *E. coli* or *S. aureus* bioparticles in the presence of soluble GST or Anxa5 for 2 h, and engulfing phagocytes were analyzed using flow cytometry (*n* = 3). **b**, **c** LR73 cells transfected with Tim-4 or Tim-4^AAA^ were incubated with FITC-labeled *E. coli* or *S. aureus* bioparticles for 2 h, stained with anti-HA antibody, and observed using confocal microscopy (**b**) or flow cytometry (**c** *n* = 3). Yellow arrows indicate Tim-4 or Tim-4^AAA^-positive cells, and white arrows indicate Tim-4 or Tim-4^AAA^-negative cells. Scale bar, 10 µm. All data are shown as the mean ± standard error of mean. Images are representative of three independent experiments. *P < 0.05, **P < 0.01. NS not significant.

We next investigated whether recognition of PS is necessary for Tim-4-mediated binding of *E. coli* or *S. aureus* particles. LR73 cells stably expressing Tim-4 were incubated with the bioparticles in the presence of purified Anxa5, a PS-binding protein. Phagocytosis of the bioparticles by the cells was unaffected by Anxa5, whereas phagocytosis of apoptotic cells was substantially impaired (Fig. 3a). We also used a Tim-4 mutant (Tim-4^AAA^), which does not bind PS and does not promote efferocytosis, to test the PS dependency of Tim-4-mediated phagocytosis of the bioparticles. LR73 cells expressing Tim-4^AAA^ were incubated with the bioparticles, and phagocytosis of the particles was analyzed using confocal microscopy or flow cytometry. Confocal microscopy results showed that both Tim-4 and Tim-4^AAA^-positive cells contained more *E. coli* or *S. aureus* particles than Tim-4-negative cells (Fig. 3b). In addition, the percentage of LR73 cells overexpressing Tim-4^AAA^ that phagocytized *E. coli* or *S. aureus* particles was comparable with phagocytosis by Tim-4- overexpressing cells. However, as expected, Tim-4^AAA^ failed to facilitate phagocytosis of apoptotic cells (Fig. 3c), suggesting that PS recognition is not necessary for Tim-4-mediated phagocytosis of *E. coli* or *S. aureus* particles. This finding is supported by the fact that PS is rarely found, and instead phosphatidylglycerol (PG) and phosphatidylethanolamine (PE) are the major phospholipid components in *E. coli* or *S. aureus* membranes^34,35^.

### The cytoplasmic tail of Tim-4 is dispensable for phagocytosis of the bioparticles

**Fig. 4:**
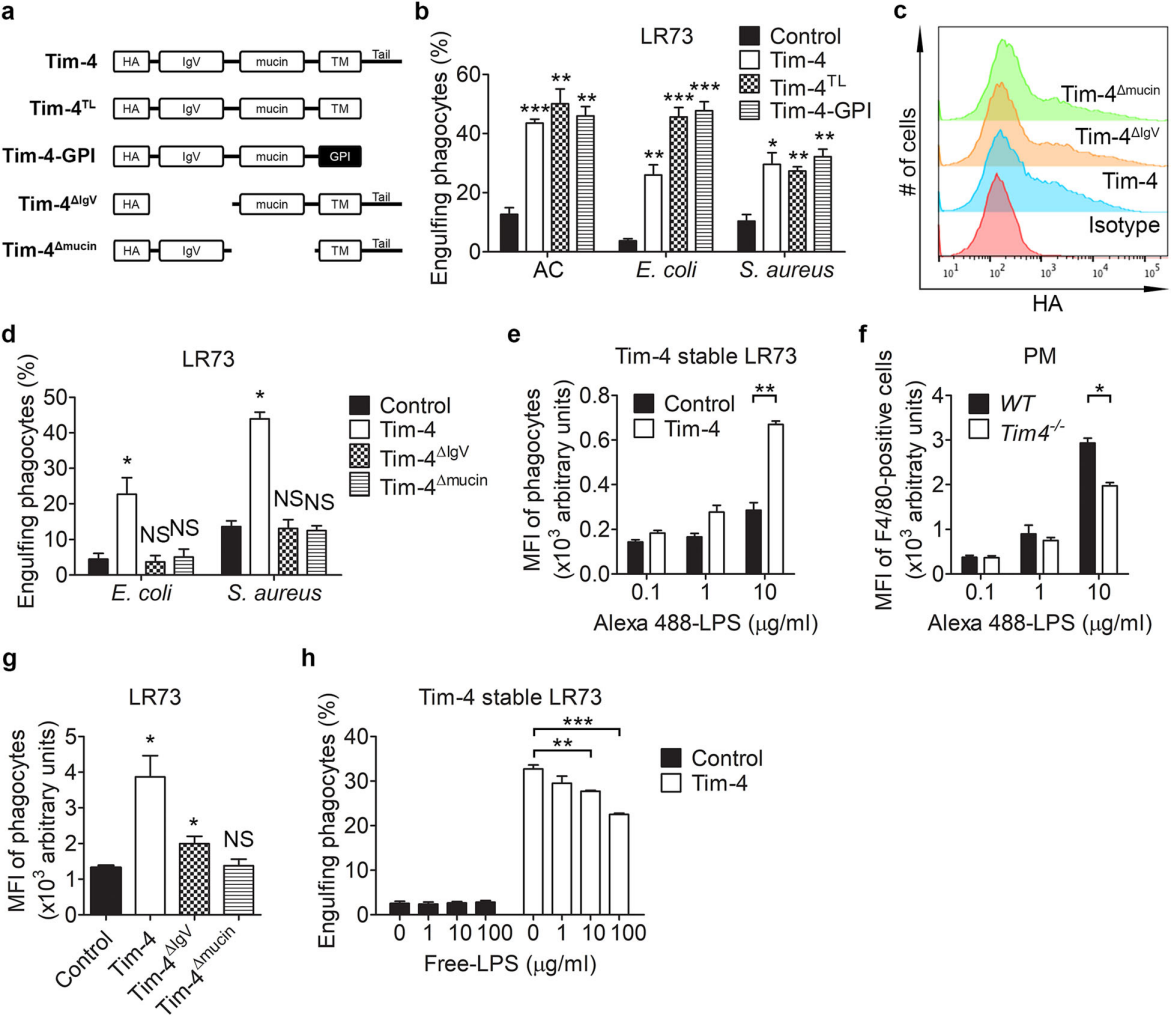
The cytoplasmic tail of Tim-4 is unnecessary for Tim-4-mediated phagocytosis of the bioparticles. **a** Schematic diagram of the constructs used in the study. HA hemagglutinin tag, GPI glycosylphosphatidylinositol, TM transmembrane. **b** LR73 cells transfected with the indicated plasmids were incubated with *E. coli* or *S. aureus* bioparticles for 2 h. Engulfing phagocytes were analyzed using flow cytometry (*n* = 3). **c** LR73 cells transfected with the indicated plasmids were stained with anti-HA antibody and analyzed using flow cytometry. **d** LR73 cells transfected with the indicated plasmids were incubated with FITC-labeled *E. coli* or *S. aureus* for 2 h, and engulfing phagocytes were analyzed using flow cytometry (*n* = 4). **e**–**g** LR73 cells stably expressing Tim-4 (**e** *n* = 3), peritoneal macrophages (**f** *n* = 3) derived from *WT* or *Tim-4*^*−/−*^ mice, or LR73 cells transfected with the indicated plasmids (**g** *n* = 3) were incubated with Alexa 488-conjugated LPS for 2 h, and the MFI of Alexa 488 fluorescence from the phagocytes was measured using flow cytometry. **h** The indicated cells were incubated with FITC-labeled *E. coli* particles in the presence of the indicated concentrations of free LPS for 2 h, and engulfing phagocytes were analyzed using flow cytometry (*n* = 3). All data are shown as the mean ± standard error of mean. Images are representative of two independent experiments. *P < 0.05, **P < 0.01, ***P < 0.001. NS not significant.

Tim-4 mutants that do not contain the cytoplasmic tail or the transmembrane domain are still able to initiate the phagocytosis of apoptotic cells^27,28^. Accordingly, the general consensus is that Tim-4 does not mediate direct signaling by itself. We wondered whether signaling in Tim-4-mediated phagocytosis of *E. coli* or *S. aureus* particles is different from that in Tim-4-mediated efferocytosis. To address this, Tim-4-GPI, which lacks both the transmembrane region and the cytoplasmic domain of Tim-4, and Tim-4^TL^, which lacks the cytoplasmic tail of Tim-4, were expressed in LR73 cells. The phagocytosis of *E. coli* or *S. aureus* particles mediated by the mutants was compared with that mediated by *WT* Tim-4. As previously reported^27^, both mutants promoted phagocytosis of apoptotic cells to the same level as that mediated by Tim-4. Similarly, both mutants robustly enhanced the phagocytosis of *E. coli* or *S. aureus* particles, which was comparable to the level of phagocytosis promoted by *WT* Tim-4 (Fig. 4a, b). These results indicate that the cytoplasmic tail and the transmembrane domain of Tim-4 are dispensable for phagocytosis of *E. coli* or *S. aureus* particles, and that signal transduction during Tim-4-mediated phagocytosis of the bioparticles could be analogous to signal transduction during Tim-4-mediated phagocytosis of apoptotic cells.

Next, we investigated which part of the extracellular region of Tim-4 is pivotal in the phagocytosis of the bioparticles. To address this, we generated the following gross deletion mutants of Tim-4: Tim-4^ΔIgV^ and Tim-4^Δmucin^ (Fig. 4a). Expression of the mutants on the cell surface was confirmed using flow cytometry (Fig. 4c), and phagocytosis of *E. coli* or *S. aureus* particles by LR73 cells overexpressing Tim-4 or the mutants was compared. Neither Tim-4^ΔIgV^ nor Tim-4^Δmucin^ promoted phagocytosis of the bioparticles (Fig. 4d), suggesting that both the IgV and the mucin domain of Tim-4 are important for the phagocytosis of the particles.

LPS is the major component of the outer membrane of Gram-negative bacteria. Because Tim-4 promotes phagocytosis of *E. coli* particles, we next tested whether Tim-4 recognizes LPS. To test this, LR73 cells stably expressing Tim-4 were incubated with Alexa 488-labeled LPS. The MFI of the fluorescence was higher in LR73 cells stably expressing Tim-4 than in control cells (Fig. 4e). By contrast, the MFI of the fluorescence was lower in peritoneal macrophages derived from *Tim-4*^*−/−*^ mice than in peritoneal macrophages derived from *WT* mice (Fig. 4f). Especially, the MFI of cells expressing Tim-4^ΔIgV^ than cells expressing Tim-4^Δmucin^ was significantly higher although the MFI of cells expressing Tim-4^ΔIgV^ was still lower than cells expressing *WT* Tim-4 (Fig. 4g). In addition, phagocytosis of *E. coli* particles was inhibited by free LPS in a dose-dependent manner (Fig. 4h), indicating that LPS on *E. coli* particles acts as a ligand for Tim-4-mediated phagocytosis of the particles. Taken together, these data suggest that Tim-4 recognizes and phagocytizes exogenous bioparticles as well as apoptotic cells, and so could function as a scavenger receptor.

## Discussion

The IgV domain of Tim-4 recognizes PS^36,37^, but the role of the mucin domain in efferocytosis is unclear. By recognizing exogenous particles, the mucin domain could be important for the function of Tim-4 as a scavenger receptor. Although the truncation mutants Tim-4^ΔIgV^ and Tim-4^Δmucin^ were expressed on the cell surface, they failed to facilitate phagocytosis of the bioparticles. This effect might be caused by misfolding of the Tim-4 mutants due to the gross deletion of the domains. However, class D scavenger receptors contain a mucin domain^1,38^. For example, CD68 serves as a scavenger receptor for oxidized LDL. CD68 consists of a short cytoplasmic tail and an extracellular region containing a mucin domain and a LAMP domain^39,40^. The size and structure of CD68 are very similar to those of Tim-4. The mucin domain of CD68 is rich in serine and threonine residues, which serve as attachment sites for carbohydrates, and are important for the activity of the scavenger receptor. Accordingly, the structural similarity between Tim-4 and CD68 suggests that the mucin domain of Tim-4 may recognize bioparticles such as *E. coli* and *S. aureus*, and that Tim-4 could function as a scavenger receptor, which is further supported by binding of LPS to the mucin domain of Tim-4 (Fig. 4g).

Based on the results of this study, we suggest that LPS is a ligand for Tim-4. LPS is present on the outer membrane of Gram-negative bacteria. However, Tim-4 also promoted the phagocytosis of *S. aureus* (Gram-positive bacteria) and zymosan A, as well as *E. coli* (Gram-negative bacteria) and Tim-4-mediated phagocytosis of *E. coli* particles was partially blocked by free LPS (Fig. 4h), suggesting that Tim-4 shows promiscuous ligand binding, which is a general characteristic of scavenger receptors^41^. The identification of further ligands for Tim-4 warrants further study.

The cytoplasmic tail of Tim-4 is dispensable for Tim-4-mediated efferocytosis. Similarly, phagocytosis of the exogenous particles mediated by a Tim-4 mutant without the cytoplasmic tail was commensurate with phagocytosis mediated by *WT* Tim-4. This result suggests that the cytoplasmic tail of Tim-4 is unnecessary for phagocytosis of the bioparticles, and that Tim-4 does not mediate direct signaling during phagocytosis of *E. coli* and *S. aureus* particles; this is analogous to the process that occurs during Tim-4-mediated efferocytosis. Notably, phagocytosis of *E. coli* particles but not apoptotic cells or *S. aureus* mediated by Tim-4^TL^ and Tim-4-GPI was higher than that mediated by Tim-4, which is statistically significant (Fig. 4b). This might be due to a difference in the expression level of the Tim-4 proteins, which could cause a difference in the avidity of the proteins for *E. coli* particles. The avidity of Tim-4^TL^ and Tim-4-GPI could be stronger than that of Tim-4. Another possibility is that the truncation of the cytoplasmic tail and/or the transmembrane domain in the mutants could cause a conformational change in the extracellular region, which increases the binding affinity for *E. coli* or exposes more of the binding site for *E. coli* particles.

Studies conducted over the past decade have aided the understanding of signal transduction in Tim-4-mediated efferocytosis. It will be intriguing to study whether signaling in Tim-4-mediated phagocytosis of the bioparticles is similar to that in Tim-4-mediated efferocytosis. The recognition of PS by Tim-4 leads to the production of anti-inflammatory mediators and provokes anti-inflammatory responses. The recognition of other ligands by Tim-4 could generate the opposite effects. Therefore, how Tim-4 elicits different responses for particular ligands, and what are the shared or unique signal transduction pathways activated by particular ligands, are important questions that need to be addressed in future studies.

Collectively, our observations suggest that Tim-4 functions not only as a PS receptor but also as a scavenger receptor for exogenous particles. Given that Tim-4 plays an important role in immune regulation and the pathogenesis of allergic diseases, these findings could be utilized toward developing a therapy to treat Tim-4-associated disorders.

## Materials and methods

### Plasmids and reagents

All plasmids in this study were constructed and sequenced to confirm their identity. The Tim-4, Tim-4^AAA^, Tim-4^TL^, Tim-4-GPI, and Anxa5-GPI plasmid used in this study have been previously described^33^. Tim-4^ΔIgV^ lacking the IgV domain of Tim-4 (residues 25–134) and Tim-4^ΔMucin^ without the mucin domain (residues 135–270) were constructed into the pDisplay vector using a PCR-based method. The antibodies used in the study were anti-HA antibody (Santa Cruz, SC-7392), PE-conjugated anti-F4/80 antibody (Biolegend, 123110), Alexa Fluor 405-conjugated anti-mouse antibody (Invitrogen, A31553), Alexa Fluor 488-conjugated anti-mouse antibody (Invitrogen, A-11029), and Alexa Fluor 555-conjugated anti-mouse antibody (Invitrogen, A-21422). Polystyrene beads (FluoSpheres carboxylate beads, Invitrogen, F8826), FITC-labeled *E. coli* (E-2861), *S. aureus* (S-2851), and zymosan A (Z-2841) bioparticles were purchased from Invitrogen. Alexa Fluor 488-conjugated LPS (L-23351) was purchased from Molecular Probes. LPS from *E. coli* O55:B5 (L2880) was purchased from Sigma Aldrich. About 6.0–8.0-μm streptavidin-coated polystyrene particles (SVP-60-5) were purchased from Spherotech. For IgG opsonization, FITC-conjugated streptavidin antibody (200-402-095) was purchased from Rockland Immunochemicals. Dexamethasone (265005) and TAMRA-SE (C1171) were purchased from Merck and ThermoFisher, respectively.

### Cell culture and transfections

293T cells were purchased from ATCC and LR73 cells were previously used^28^. LR73 cells were cultured in alpha-MEM containing 10% FBS and 1% penicillin–streptomycin–glutamine. LR73 cells were transfected with the indicated plasmids using Lipofectamine 2000 (Invitrogen) according to the manufacturer’s protocol. For LR73 cells stably expressing HA-Tim-4, LR73 cells were co-transfected with HA-Tim-4 and pApuro and maintained in the presence of puromycin for 1 month. Then, LR73 cells expressing HA-Tim-4 on the cell surface were selected using flow cytometry. All cell lines used in the study were negative for mycoplasma.

### Mice

Tim-4^*−/−*^ mice (RBRC04895) were obtained from Riken BioResource Center (Japan) and C57BL/6 mice were purchased from Taconic bioscience. Mice, 8–10-weeks old, were used regardless of sex in the study. For mouse studies, no statistical methods were used for sample estimate, no randomization was used, and no blinding was done. All experiments using mice were approved by the animal care and ethics committees of the Gwangju institute of science and technology (GIST) in accordance with the national institutes of health guide for the care and use of laboratory animals.

### FRET analysis of Rac1 activity

Raichu-Rac1 2248x plasmid was a gift from Michiyuki Matsuda (Kyoto University). LR73 cells stably expressing HA-Tim-4 or empty vector were plated on 35-mm confocal dishes and transfected with Raichu-Rac1 2248x plasmid. Twenty-four hours after transfection, FRET images of basal level of Rac1 were obtained with the Ratio Imaging method equipped with Olympus FV1000 SPD (Olympus, Tokyo, Japan). For FRET ratio calculation, CFP and YFP images were subtracted with the respective background intensity calculated by the mean-intensity value of pixels inside background mask. The FRET ratio was calculated as the ratio between processed YFP and CFP images. FRET ratio was presented as a heatmap by mapping FRET ratio to color and total intensity to opacity. All processing was performed using custom code of MATLAB R2019b and image-processing toolbox.

### Phagocytosis assay

For phagocytosis assay, LR73 cells were plated on 24-well culture dish and transfected with the indicated plasmids. Twenty-four hours after transfection, the cells were incubated with FITC-labeled *E. coli*, *S. aureus*, or zymosan A bioparticles for 2 h at 37 °C. The cells then were extensively washed with ice-cold PBS, trypsinized, and stained with anti-HA antibody (1:50) and Alexa Fluor 555-conjugated anti-mouse antibody (1:2000) or Alexa Flour 488-conjugated anti-mouse antibody (1:2000) for 30 min at 4 °C. Then, engulfing phagocytes were analyzed using flow cytometry. HA- and FITC-positive cells were considered as phagocytes engulfing the targets. For binding assay, LR73 cells stably expressing Tim-4 were trypsinized, suspended with alpha-MEM, and incubated with FITC-labeled *E. coli* or *S. aureus* bioparticles for 2 h at 4 °C on a rotator. Then, the cells were washed with ice-cold PBS and analyzed using flow cytometry. For in vivo phagocytosis assay, FITC-labeled *E. coli* or *S. aureus* bioparticles were injected into the peritoneum of *WT* or *Tim-4*^*−/−*^ mice. Twenty minutes after injection, the mice were sacrificed, and peritoneal exudates were stained with PE-conjugated anti-F4/80 antibody (1:100) and analyzed using flow cytometry. FITC- and F4/80-positive cells were considered as peritoneal macrophages engulfing the targets.

About 6-μm streptavidin beads were incubated with FITC-conjugated anti-streptavidin antibody for 30 min. LR73 cells stably expressing Tim-4 were incubated with IgG-opsonized beads for 2 h and analyzed by flow cytometry. To block phagocytosis of *E. coli* particles, LR73 cells were preincubated with free LPS for 30 min, then incubated with FITC-labeled *E. coli* particles for 30 min, and analyzed by flow cytometry.

### Efferocytosis assay

LR73 cells stably expressing HA-Tim-4 or LR73 cells transiently transfected with the indicated plasmids were incubated with TAMRA-labeled apoptotic thymocytes. Two hours after incubation, the phagocytes were extensively washed with ice-cold PBS. Then, the cells were trypsinized, stained with anti-HA antibody and Alexa Fluor 488-conjugated anti-mouse antibody, and analyzed using flow cytometry. Double-positive cells for HA and TAMRA were considered as phagocytes engulfing apoptotic thymocytes. TAMRA-labeled apoptotic cells were generated as previously described^42^. Briefly, thymocytes derived from 4- to 8-week-old C57BL/6 mice were stained with 25 μM of TAMRA-SE for 20 min and washed with RPMI medium containing 10% serum. Then, apoptosis in thymocytes was induced using 50 μM of dexamethasone in an incubator with 5% CO_2_ at 37 ^o^C for 4 h. The thymocytes were washed with PBS and resuspended with alpha-MEM or DMEM medium.

### LPS-binding assay

LR73 cells stably expressing Tim-4 or transfected with the indicated plasmids were incubated with 10 μg/ml of Alexa Fluor 488-conjugated LPS for 2 h, washed with ice-cold PBS, trypsinized, stained with anti-HA antibody and Alexa Fluor 405-conjugated anti-mouse antibody, and analyzed by flow cytometry.

### Immunofluorescence staining

LR73 cells were plated on 35-mm confocal dishes and transfected with the indicated plasmids. One day after transfection, the cells were incubated with FITC-labeled bioparticles or TAMRA-stained apoptotic thymocytes for 2 h at 37 °C (4 °C for the binding assay). The cells were then washed with ice-cold PBS, fixed with 4% paraformaldehyde in PBS for 15 min, permeabilized with 0.1% Triton X-100 for 5 min, and blocked with 10% BSA for 30 min. Next, the cells were incubated with anti-HA antibody (1:50) in PBS with 3% BSA at 4 °C overnight, and then stained with Alexa Fluor 555-conjugated anti-mouse antibody (1:2000) or Alexa Fluor 488-conjugated anti-mouse antibody (1:2000) for 1 h. After that, the cells were stained with Hoechst 33342 (Invitrogen) for 10 min. Images were acquired using Zeiss LSM 700 (Oberkochen, Germany) or Olympus FV1000 SPD (Olympus, Tokyo, Japan). For peritoneal macrophage staining, the whole procedure was the same as that of LR73, but the cells instead were incubated with the targets for 20 min and stained with PE-conjugated anti-F4/80 (1:100).

### Statistical analysis

Each experiment was independently performed at least two times, and all data are shown as the mean ± standard error of mean. Unpaired Student’s *t* test or one-way ANOVA was used to analyze statistical differences, which were calculated using the GraphPad Prism 6 software (GraphPad, La Jolla, CA, USA). Statistical significance was accepted when *P* values were <0.05.
